# Supplementary material for: Effect of a Novel Trivalent Vaccine Formulation against Acute Lung Injury Caused by Pseudomonas aeruginosa
Source: Vaccines (Basel). 2023 Jun 11;11(6):1088. doi: 10.3390/vaccines11061088 (PMC10304393; doi:10.3390/vaccines11061088)
Supplement: Supplementary file 1 [file vaccines-11-01088-s001.zip › vaccines-2405212-supplementary.pdf]

Table S1. PCR primer sets

PCR product	name	sequence
PcrV	PcrV5-*Sph*I	5'-G CAT GCG AAA ATG GAA GTC AGA AA-3'
	PcrV3-*Hind*III	5'-A AGC TTG cta GAT CGC GCT GAG AA-3'
OprF$_{\#190-\#342}$	OprF5-190-*Sph*I-TG	5'-G CAT GCG AAG GCT CCG GCT CCG GAA CC-3'
	OprF3-342-*Hind*III	5'-AAG CTT tta TTC AAC GCG ACG GTT GAT AG-3'
ToxA$_{\#406-\#613(E553\Delta)}$	tox103_CT5-*Sph*I_TG	5'- G CAT GCG AAA GAC GTC AGC TTC AGC ACC-3'
	toxCT_3-*Hind*III	5'- AAG CTT tta CTT CAG GTC CTC GCG-3'
	toxA_D553	5'- C GAG AAT GGT [ΔCTC] CAG GCG CCC GCC-3'
POmT		
PcrV domain	PcrV5-*Sph*I	5'-G CAT GCG AAA ATG GAA GTC AGA AA-3'
	PcrV3 (no stop codon)	5'-GAT CGC GCT GAG AAT GTC GCG-3'
	PcrV3-GGGGS	5'-GGA TCC GCC ACC GCC GAT CGC GCT GAG AAT GTC GCG-3'
OprF$_{\#190-\#342}$ domain	OprF190-5	5'-GCT CCG GCT CCG GAA CCG-3'
	OprF342-3	5'-C TTC AAC GCG ACG GTT GAT AGC GC-3'
	OprF190-342-GGGGS-5	5'-GGC GGT GGC GGA TCC GCT CCG GCT CCG GAA CCG-3'
	OprF190-342-GSGGSG-3	5'-ACC GGA ACC CCC TGA ACC TTC AAC GCG ACG GTT GAT AGC GC-3'
ToxA$_{\#406-\#613(E553\Delta)}$ domain	tox103_CT5	5'-GAC GTC AGC TTC AGC ACC CGC G-3'
	tox103_CT5- GSGGSG	5'-GGT TCA GGG GGT TCC GGT GAC GTC AGC TTC AGC ACC CGC G-3'
	toxCT_3-*Hind*III	5'-AAG CTT tta CTT CAG GTC CTC GCG-3'

PcrV, full-length PcrV; OprF, the outer membrane domain (#190–342) of OprF; mTOX, a non-catalytic mutated domain (#406–613, E553Δ) of exotoxin A; POmT, a conjugate of PcrV, and parts of OprF and mutated exotoxin A.
